# ZIP9 mediates the effects of DHT on learning, memory and hippocampal synaptic plasticity of male Tfm and APP/PS1 mice

**DOI:** 10.3389/fendo.2023.1139874

**Published:** 2023-05-25

**Authors:** Leigang Song, Huan Chen, Dan Qiao, Bohan Zhang, Fangzhen Guo, Yizhou Zhang, Chang Wang, Sha Li, Huixian Cui

**Affiliations:** ^1^ Department of Human Anatomy, Hebei Medical University, Shijiazhuang, Hebei, China; ^2^ Department of Sports Human Science, Hebei Sport University, Shijiazhuang, Hebei, China; ^3^ Neuroscience Research Center, Hebei Medical University, Shijiazhuang, Hebei, China; ^4^ Hebei Key Laboratory of Neurodegenerative Disease Mechanism, Hebei Medical University, Shijiazhuang, Hebei, China

**Keywords:** androgen, ZIP9, hippocampus, synaptic plasticity, learning and memory

## Abstract

Androgens are closely associated with functions of hippocampal learning, memory, and synaptic plasticity. The zinc transporter ZIP9 (SLC39A9) regulates androgen effects as a binding site distinct from the androgen receptor (AR). However, it is still unclear whether androgens regulate their functions in hippocampus of mice through ZIP9. Compared with wild-type (WT) male mice, we found that AR-deficient male testicular feminization mutation (Tfm) mice with low androgen levels had learning and memory impairment, decreased expression of hippocampal synaptic proteins PSD95, drebrin, SYP, and dendritic spine density. Dihydrotestosterone (DHT) supplementation significantly improved these conditions in Tfm male mice, although the beneficial effects disappeared after hippocampal ZIP9 knockdown. To explore the underlying mechanism, we first detected the phosphorylation of ERK1/2 and eIF4E in the hippocampus and found that it was lower in Tfm male mice than in WT male mice, it upregulated with DHT supplementation, and it downregulated after hippocampal ZIP9 knockdown. Next, we found that the expression of PSD95, p-ERK1/2, and p-eIF4E increased in DHT-treated mouse hippocampal neuron HT22 cells, and ZIP9 knockdown or overexpression inhibited or further enhanced these effects. Using the ERK1/2 specific inhibitor SCH772984 and eIF4E specific inhibitor eFT508, we found that DHT activated ERK1/2 through ZIP9, resulting in eIF4E phosphorylation, thus promoting PSD95 protein expression in HT22 cells. Finally, we found that ZIP9 mediated the effects of DHT on the expression of synaptic proteins PSD95, drebrin, SYP, and dendritic spine density in the hippocampus of APP/PS1 mice through the ERK1/2-eIF4E pathway and affected learning and memory. This study demonstrated that androgen affected learning and memory in mice through ZIP9, providing new experimental evidence for improvement in learning and memory in Alzheimer’s disease with androgen supplementation.

## Introduction

Androgens are steroid hormones synthesized in the gonads, adrenal glands, and brain (1) and are important for the development of male sexual organs, secondary sexual characteristics, sexual desire, and normal sexual function. They also regulate learning and memory in the hippocampus by maintaining normal synaptic plasticity and regulating synaptic plasticity-related proteins and dendritic spine density (2–5).

Alzheimer’s disease (AD) is a neurodegenerative disease characterized by progressive memory impairment and cognitive impairment (6), with pathological changes that include formation of senile plaques due to excessive deposition of amyloid β (Aβ), neurofibrillary tangles, and extensive neuronal loss (7). There is an increased risk of AD caused by low serum androgen levels (8–11) or anti-androgen therapy (12–14). While androgen supplementation has been found to improve memory impairment (15, 16), the neuroprotective mechanism of androgens on learning and memory in AD remains unclear.

Testicular feminization mutation (Tfm) mice are androgen receptor (AR)-deficient (17) and have decreased circulating androgen (18) due to hereditary single-base deletion of the X chromosome. Surprisingly, learning and memory in Tfm male mice was significantly improved by androgen supplementation. This suggests that there may be androgen-binding sites other than the classical AR that mediate androgen-rescuing learning and memory. The ZIP9 (Zrt-, Irt-like protein family solute carrier family 39 member 9, SLC39A9) is the ninth in a family of 14 ZIP proteins. It has seven transmembrane domains and one intracellular C-terminal domain and is responsible for transmembrane transport of Zn^2+^. It is also the only ZIP protein that can couple with G-proteins during signal transduction across membranes (19, 20). Recent studies have found that ZIP9 can mediate biological effects of androgens in a variety of cell types. Some of these effects include proliferation of human and mouse melanoma cells (21), migration of human prostate (22) and bladder (23) cancer cells, apoptosis of human breast cancer and prostate cancer cells (20, 24, 25), and the expression of tight junction proteins in mouse (26) and rat (27) Sertoli cells. In a previous study it was found that androgens induced the interaction between ZIP9 and Gnα11, which affected the expression of postsynaptic density protein 95 (PSD95) in mouse hippocampal neuron HT22 cells (28). However, it is not clear whether ZIP9 mediates the effects of androgens on learning and memory in Tfm mice.

In this study, we explored whether androgens induced by ZIP9 affected learning, memory and hippocampal synaptic plasticity in Tfm male mice and investigated the underlying mechanism. Further, we examined whether learning and memory of AD animal model-APP/PS1 mice was mediated through this mechanism.

## Materials and methods

### Animals

Female Tfm and male C57BL/6J mice were purchased from Jackson Laboratory (Stock #000569, BarHarbor, ME, USA) and Vital River Laboratory Animal Technology Co., Ltd (Beijing, China) respectively. We induced mating of Tfm female with male C57BL/6J mice which produced the following offspring types: wild-type (WT) female, WT male, Tfm female, and Tfm male. All offspring were genotyped using real-time polymerase chain reaction (PCR), and only WT and Tfm male mice were selected for this study. APP/PS1 mice were provided by the Vital River Laboratory Animal Technology Co., Ltd. (Beijing, China). The mice were raised and bred at the Experimental Animal Research and Service Center of Hebei Medical University under conditions of constant temperature (22 ± 2°C), constant humidity (55 ± 5%), lighting (12-h light/dark cycle), and free access to food and water. All animal experiments were carried out according to the National Institutes of Health Guide for Care and Use of Laboratory Animals and approved by Laboratory Animal Ethical and Welfare Committee of Hebei Medical University.

### Cell culture

Mouse hippocampal neuron HT22 cells were cultured in phenol red-free DMEM/F12 medium (cat# PM150316, Procell, China) containing 10% fetal bovine serum (FBS) and 1% penicillin-streptomycin under conditions (37 °C, 5% CO_2_) in a humidified atmosphere. After digestion with 0.25% trypsin at 85%-90% confluency, the cells were seeded into a 6-well plate, and virus infection experiments were carried out to establish ZIP9 knockdown or overexpression HT22 stable cell lines. Subsequently, the cells were transferred into 6 or 24-well plates for Western blot and immunofluorescence staining. With or without pretreatment with 100 nM ERK1/2 inhibitor SCH772984 (cat#: S7101 Selleck, USA) or 25 nM eIF4E inhibitor eFT508 (eFT, cat#: HY-100022, MCE, USA) for 2 h, the cells in the experimental groups were treated with 10 nM dihydrotestosterone (DHT, cat#: A0462, Tokyo Chemical Industry, Japan) for 24 h, and those in the control group were treated with an equal volume of dimethyl sulfoxide (DMSO).

### Establishment of ZIP9 knockdown or overexpression HT22 stable cell lines

ZIP9-knockdown and ZIP9-overexpress lentivirus targeting ZIP9 (5’ -ATTGTGTTCGTGGCAATAA-3’) and the corresponding negative control lentivirus were provided by Genechem Inc. (Shanghai, China). HT22 cells were infected with the lentivirus for 12 h at 20-30% confluency. The HT22 cells were then cultured with fresh medium for 72 h until 4.5µg/mL puromycin was added for 48 h to kill uninfected cells. Stable clones were selected using 2.25 µg/mL puromycin after cell passaging, and stable ZIP9 knockdown or overexpression HT22 cell lines were established.

### Immunofluorescence staining

HT22 cells were fixed with 4% paraformaldehyde (PFA) for 15 min, blocked with 10% donkey serum for 1 h at room temperature, and incubated overnight at 4°C with the following primary antibodies: rabbit anti-PSD95 (cat#: ab18258, Abcam, USA), rabbit anti-phospho-ERK1/2 (cat#: 9101, cell signaling, USA), and rabbit anti-phospho-eIF4E (cat#: ab76256, Abcam, USA). The next day, the cells were incubated with donkey anti-rabbit fluorescent secondary antibody (cat#: A21207, Invitrogen, USA) for 2 h at room temperature in the dark. They were then counterstained with 4’,6-diamidino-2-phenylindole (DAPI) (cat#: C0065, Solarbio, China) for 10 min and sealed with anti-fluorescence quenching sealing tablets (cat#: S2100, Solarbio, China). Images were taken using a laser confocal microscope (Olympus, Japan), and the average optical density was analyzed using Fiji software (National Institutes of Health, USA). Intra and inter-assay coefficients of variation were 2.44%-4.95% and 4.07%-9.81% respectively.

### Castration of APP/PS1 mice

After anesthetizing 6-month-old APP/PS1 mice with isoflurane (cat#: R510-22-10, RWD, China), small incisions were made in the scrotums to remove the testes in the experimental group, while the scrotums were cut open and sutured without hurting the testes in the sham operation group.

### Adeno-associated virus and microinjection in CA1

Adeno-associated virus (AAV9) and negative control virus with GV478 as a vector were provided by Genechem Inc. (Shanghai, China). The mice were anesthetized with isoflurane and fixed on a brain stereotaxic instrument (StereoDrive, NeuroStar, Germany) lying prone. After the anterior and posterior fontanelle were fully exposed, the CA1 region (left: ML = -2.29 mm, AP = -2.28 mm, DV = 1.62 mm; right: ML = 2.29 mm, AP=-2.28 mm, DV = 1.62 mm) was located by the mouse brain atlas (Watson, 3rd edition). Through the microinjection system, 1μL (1×10^13^ v.g./ml) of virus was injected into the bilateral CA1 region.

### Grouping and administration of mice

All our animal experiments followed the 3R principle, that is, replacement, reduction and refinement. According to the experimental design, we calculated the number of animals needed for the experiments in advance, which not only ensured that the sample size met the needs of the experiments, but also avoided unnecessary wastage of resources and mice. Using the formula provided by Charan et al. (29), we determined that the number of mice in each group was 12. Twelve three-month-old WT male mice were in WT+nc-RNAi group, and 36 Tfm male mice were equally divided between Tfm+nc-RNAi, Tfm+nc-RNAi+DHT, and Tfm+ZIP9-RNAi+DHT groups. Four weeks after virus injection, the Tfm+nc-RNAi+DHT and Tfm+ZIP9RNAi+DHT groups were injected intraperitoneally with physiological DHT (1 mg/kg body weight), and the WT+nc-RNAi and Tfm+nc-RNAi groups were injected with an equal volume of vehicle until behavioral tests were completed. Forty-eight 6-month-old APP/PS1 mice were divided equally into Sham+nc-RNAi, Cast+nc-RNAi, Cast+nc-RNAi+DHT, and Cast+ZIP9-RNAi+DHT groups, and then they were castrated or sham operated. Four weeks later, the mice were injected with adeno-associated virus. Four weeks after virus injection, the Cast+nc-RNAi+DHT and Cast+ZIP9-RNAi+DHT groups were injected intraperitoneally with DHT, while the Sham+nc-RNAi and Cast+nc-RNAi groups were injected intraperitoneally with an equal volume of vehicle until the behavioral test was completed. The average weight of mice was 27-30g. All the mice were subjected to behavioral tests, and the behavioral data of 10 mice in each group was used for statistical analysis.

### Y-maze

Testing was performed using a Y-shaped maze with arms oriented at 120° angles from each other. Each arm had a size of 30×8×15 cm with markers of different colors and shapes on the inner wall that acted as spatial localization reference for the mice. The three arms were randomly set as the novel arm, start arm, and other arm. During the training session, each mouse was placed in the start arm and allowed to explore the maze freely for 5 min with the novel arm closed off. Four hours later, the novel arm was opened during the test session, and the mouse was placed in the start arm and allowed to freely explore the three arms for 5 min. To avoid smell clues affecting the next mouse, 75% ethanol was used to wipe the bottom and inner walls of the maze after each test. The entire process of the experiment was recorded using a camera and analyzed using the SMART video tracking system. The evaluation indexes were time (%), distance (%) in the novel arm, and the number of entries into the novel arm.

### Novel object recognition test

A 3-day new object recognition experiment was performed in an open field box measuring 50×50×40 cm. On day 1, the mice were placed in a box for 5 min of adaptive training with free exploration, facing the sidewall close to the experimenter. On day 2, two identical objects were placed in the left and right corners away from the experimenter and 10 cm from each sidewall. Mice were placed in the box with their backs to the objects and allowed to explore freely for 10 min. On day 3, one of the original objects was replaced with a novel object of different color and shape, but having the same volume. The exploration time of the mice for the two objects was recorded within 10 min, and the exploration distance was 2-3 cm from the objects. Exploration behavior included placing the front paw on the object, smelling the object, licking the object. Holding a pose or climbing an object without moving is not an exploration of that object. The discrimination index represented by the ratio of exploration time of the novel object to the total exploration time, was calculated.

### Morris water maze

The Morris water maze test was used to detect spatial learning and memory in the mice. The circular pool (diameter 120 cm) was divided into four quadrants, and the platform (diameter, 6 cm) was placed in any quadrant 1 cm underwater. In the orientation navigation trial, the mice were placed into the water facing the pool wall from four quadrants, and the time to find the platform was within 60 s, that is, the escape latency. If they could not find the platform in 60 s, the mice were guided to the platform and remained there for 15 s, and the escape latency was recorded as 60 s. The distance before finding the platform and swimming trajectories were recorded. On the 6th day, a spatial probe trial was performed. After the platform was removed, mice were placed in water. The number of mice crossing the position of the platform within 60s and the time spent in the target quadrant were recorded.

### Western blot

Radioimmunoprecipitation assay lysate containing phenylmethylsulfonyl fluoride and phosphatase inhibitors was added to the samples, and proteins were extracted for quantitative analysis. After denaturation, 25 μg of protein was loaded onto a 10% sodium dodecyl sulphatepolyacrylamide gel for protein separation and electrotransferred onto polyvinylidene fluoride membranes. The membranes were blocked with 5% non-fat milk at room temperature for 1 h and incubated overnight at 4 °C with the following primary antibodies: rabbit anti-PSD95 (cat#: ab18258, Abcam, USA), rabbit anti-phospho-eIF4E (cat#: ab76256, Abcam, USA), mouse anti-eIF4E (total) (cat#: ab171091, Abcam, USA), rabbit anti-GAPDH (cat#: ab9485, Abcam, USA), rabbit anti-phospho-ERK1/2 (cat#: 9101,cell signaling, USA), mouse anti-Erk1/2 (cat#: 9107, cell signaling, USA), rabbit anti-ZIP9 antibody (cat#: GTX31817,GeneTex, USA), rabbit anti-synaptophysin (SYP cat#: CY5273, Abways, China), and rabbit anti-drebrin (cat#: 10260-1-AP, Proteintech, USA). The membranes were then incubated with goat anti-rabbit fluorescent secondary antibody (cat#611145002, Rockland, USA) or goat anti-mouse secondary antibody (cat#610144002, Rockland, USA) in dark incubation boxes for 2 h. Finally, an Odyssey imaging system (LICOR, USA) was used for visualization and analysis. The relative expression of the target protein was calculated according to the gray value of β-actin or GAPDH as a reference, and the phosphorylation level of the proteins was determined by the ratio of phosphorylated proteins to total proteins. Intra and inter-assay coefficients of variation were 1.86% to 4.73% and 4.28% to 9.77% respectively.

### Immunohistochemical staining

After the behavioral study, mice were deeply anesthetized with isoflurane, perfused with PBS, and fixed with 4% PFA. Their brains were removed and fixed in 4% PFA for 24 h. Brains were cut from the superior colliculus to the optic chiasma and separated along the median sagittal plane. The left parts were prepared for Golgi staining, and the right parts were routinely dehydrated, waxed, embedded, and cut into 5 μm-thick sections. After dewaxing, hydration, high pressure antigen repair, and blocking, the sections were incubated at 4 °C overnight with the following primary antibodies: rabbit anti-PSD95 (cat#: ab18258, Abcam, USA), rabbit anti-synaptophysin (cat#: CY5273, Abcam, China), rabbit anti-drebrin (cat#: 10260-1-AP, Proteintech, USA). Subsequently, the sections were incubated with goat anti-rabbit IgG polymer labeled with biotin (cat#: SP-9001, ZSGB-BIO, China) for 30 min, horseradish enzyme-labeled streptomycin for 1 h, and DAB staining. The hippocampal CA1 region was observed and imaged under a 40× light microscope (Leica, Germany), and the average optical density was analyzed using Fiji software (National Institutes of Health, USA). Intra-assay coefficients of variation were 2.03% to 4.89%.

### Golgi staining

Golgi staining was performed according to the protocol provided in the Golgi staining kit (cat#: GMS80020.1, GENMED, China). After 24 h of post-fixation, the left brain was immersed in mordant (Reagent A and Reagent B were mixed at 1:1) and stained for 14 days at room temperature in the dark. They were then placed in 30% sucrose solution, dehydrated at 4 °C for 48 h, and cut into 100-μm sections with oscillating tissue slicers. The sections were incubated with staining solution at room temperature for 30 min, followed by incubation with chromogenic solution for 20 min at room temperature in the dark. They were then dehydrated, made transparent, and sealed with neutral resin. The secondary or tertiary dendritic spines of apical dendrites in the hippocampal CA1 region were observed and imaged under a 100× light microscope (Olympus, Japan). Dendritic spine density was analyzed using Fiji software. Three sections were selected from each mouse and three neurons were selected from each section. Intra-assay coefficients of variation were 2.08% to 4.55%.

### Statistical analysis

SPSS26.0 statistical software was used for analysis. The results are expressed as mean ± standard deviation (SD). The Shapiro-Wilk test for normality was performed, and the Student’s t-test was used for two-sample comparisons of normally distributed data (*P* > 0.1). Levene’s test for homogeneity of variance was conducted on data from multiple groups. One-way analysis of variance (ANOVA) was performed for data with a normal distribution (*P* > 0.1) and homogeneity of variance (*P* > 0.1), and *post hoc* multiple comparisons were performed using the LSD test. The Kruskal-Wallis H test was used to compare multiple groups of quantitative data with non-normal distribution (*P* < 0.1), and *post hoc* multiple comparisons were performed with the independent-samples Kruskal-Wallis Test. The significant differences in escape latency at 1-5 days were assessed using two-way repeated-measures ANOVA. Differences were considered significant at *P* < 0.05. To ensure validity, all the data was analyzed *post-hoc* with G*Power (www.gpower.hhu.de), and the statistical power was set at equal to or greater than to 0.8.

## Results

### Effects of DHT on learning and memory in Tfm male mice after hippocampal ZIP9 knockdown

**Figure 1:**
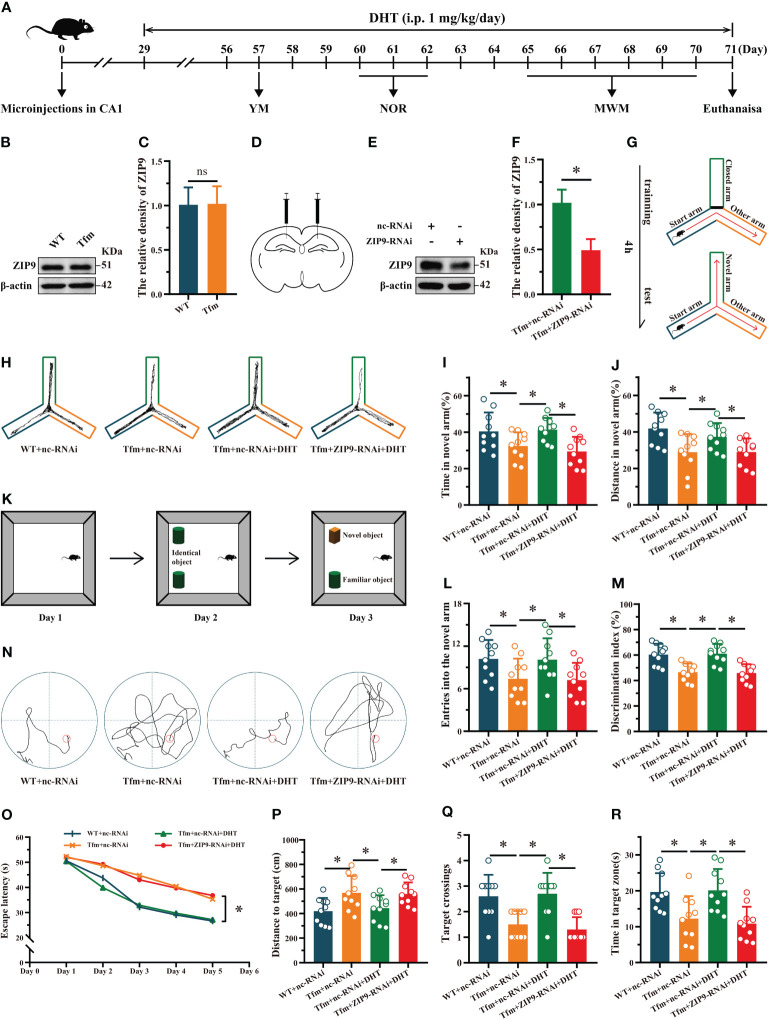
Effects of DHT on learning and memory of Tfm male mice after hippocampal ZIP9 knockdown. **(A)** Experimental procedure. Mice treated with microinjections in CA1, DHT (i.p. 1 mg/kg/day), and behavioral tests. **(B, C)** Representative Western blot **(B)** and quantification **(C)** of ZIP9 in the hippocampus of WT and Tfm male mice (*P > 0.05, n = 6). **(D)** Schematic diagram of microinjections in the hippocampal CA1 region **(E, F)**. Knockdown efficiency of ZIP9 protein in Tfm male mice hippocampi infected with AAV9-ZIP9-RNAi or AAV9-nc-RNAi (*P < 0.05, n = 6). **(G)** Schematic diagram of the YM. **(H)** Trajectories of the YM. **(I, J, L)** YM performed to assess spatial reference memory. **(K)** Schematic diagram of the NOR. **(M)** NOR performed to assess memory retention. **(N)** Trajectories of the MWM (the 5th day). **(O-R)** MWM was used to test spatial learning and memory. DHT, dihydrotestosterone; ZIP9, Zrt-, Irt-like protein 9; Tfm, Testicular feminization mutation; YM, Y-maze test; NOR, novel object recognition test; MWM, Morris water maze. (*P < 0.05, n = 10).

Western blot results showed that ZIP9 was expressed in the hippocampi of WT and Tfm male mice, and there was no significant difference between the two groups (*t*_(10)_ = -0.084, *P* = 0.935, Cohen’s *d* = 0.05) (**Figures 1B, C** **)**. To determine whether androgen affects the learning and memory of Tfm male mice through ZIP9, we transfected hippocampal neurons with AAV9-ZIP9-RNAi and control virus (AAV9-nc-RNAi). The expression of hippocampal ZIP9 in the Tfm+ZIP9-RNAi group was significantly lower than that in the Tfm+nc-RNAi group (*t*_(10)_ = 6.746, *P* < 0.05, Cohen’s *d* = 3.89) (**Figures 1D–F** **)**. We tested the effect of DHT induced by ZIP9 on the behavior of WT and Tfm male mice (**Figure 1A**).

The results of the YM showed that there were significant differences in the percentage of time spent in the novel arm (F _(3,36)_ = 5.059, *P* < 0.05, η^2^ = 0.297), the percentage of distance in the novel arm (F _(3,36)_ = 5.790, *P* < 0.05, η^2^ = 0.325), and the number of entries into the novel arm (F _(3,36)_ = 3.614, *P* < 0.05, η^2^ = 0.231) among all groups. The percentage of time spent in the novel arm, the percentage of distance in the novel arm, and the number of entries into the novel arm of the Tfm+nc-RNAi group were significantly lower than those of the WT+nc-RNAi and Tfm+nc-RNAi+DHT groups, whereas those of the Tfm+nc-RNAi+DHT group were higher than those of the Tfm+ZIP9-RNAi+DHT group (**Figures 1G-J, L** **)**.

The NOR test showed significant differences in discrimination index (DI) among all groups (F _(3,36)_ = 12.547, *P* < 0.05, η^2^ = 0.511). The DI in the Tfm+nc-RNAi group was significantly lower than that in the WT+nc-RNAi group. DHT supplementation increased the DI of Tfm male mice, whereas the increase induced by DHT disappeared after hippocampal ZIP9 knockdown (**Figures 1K, M** **)**.

In the Morris water navigation task, there were significant differences in the escape latency at 1-5 days (F_(3,36)_ = 3.502, *P* < 0.05, η^2^ = 0.203) and the distance to the target on the 5th day (F_(3,36)_ = 4.657, *P* < 0.05, η^2^ = 0.280). The values of above parameters for the Tfm+nc-RNAi group were significantly higher than those for the WT+nc-RNAi and the Tfm+nc-RNAi+DHT groups, whereas the values for Tfm+nc-RNAi+DHT group were lower than those for the Tfm+ZIP9-RNAi+DHT group. The subsequent spatial probe trial revealed differences in the number of target crossings (H = 19.807, *P* < 0.05) and time in the target zone (F_(3,36)_ = 7.598, *P* < 0.05, η^2^ = 0.388). The number of target crossings and time in the target zone of the Tfm+nc-RNAi group were lower than those of the WT+ncRNAi and Tfm+nc-RNAi+DHT groups, whereas those of the Tfm+nc-RNAi+DHT group were higher than those of the Tfm+ZIP9-RNAi+DHT group (**Figures 1N-R**).

### Effects of DHT on hippocampal PSD95, drebrin, SYP protein and dendritic spine density in Tfm male mice after hippocampal ZIP9 knockdown

**Figure 2:**
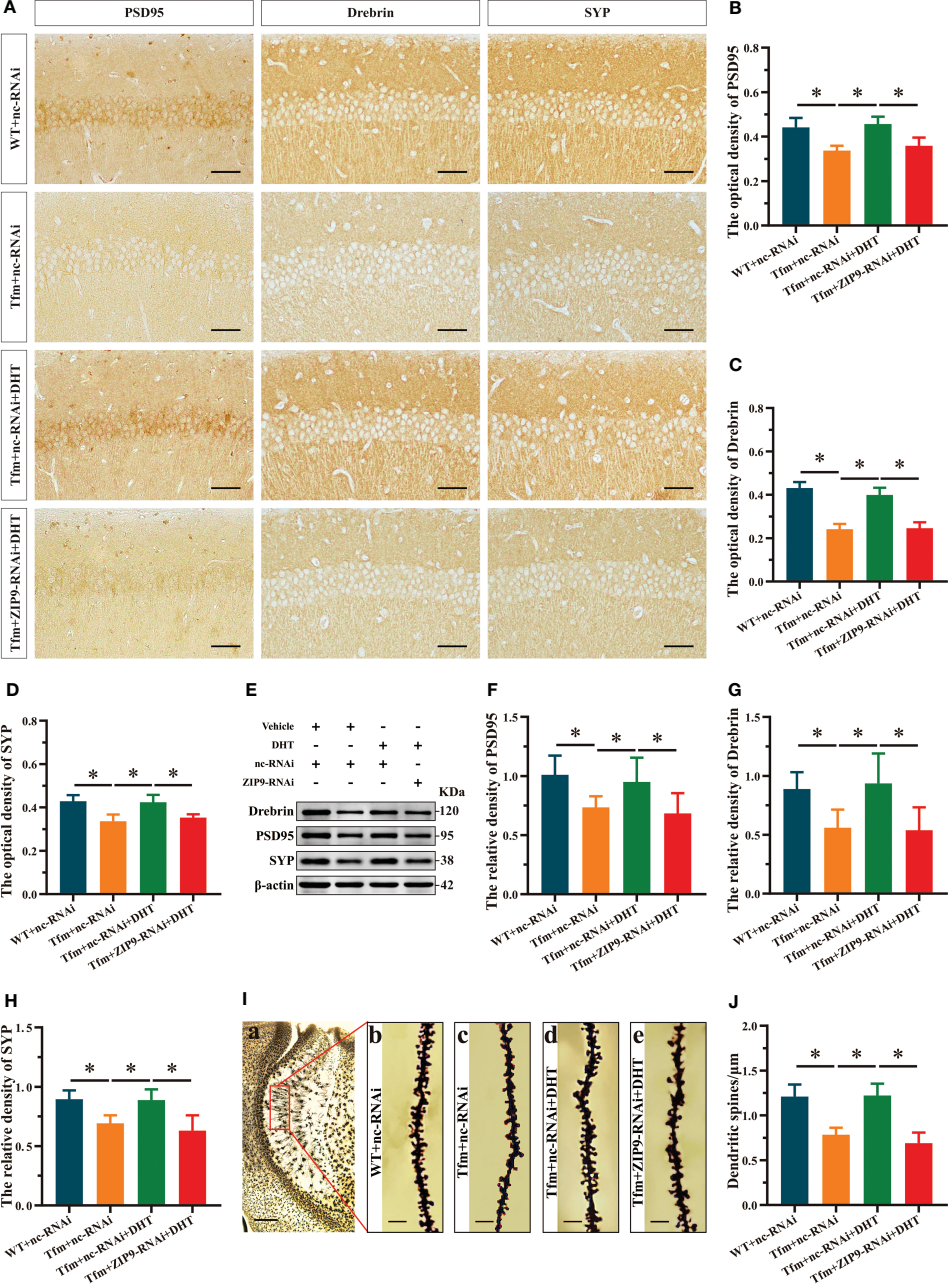
Effects of DHT on hippocampal PSD95, drebrin, SYP protein and dendrite spine density of Tfm male mice after hippocampal ZIP9 knockdown. **(A–D)** Representative immunohistochemical staining images **(A)** and quantification **(B-D)** of PSD95 **(B)**, drebrin **(C)**, and SYP **(D)** in the hippocampal CA1 region of the four groups of mice. Scale bars = 50 μm. **(E–H)** Representative Western blot **(E)** and quantification **(F–H)** of PSD95 **(F)**, drebrin **(G)**, and SYP **(H)** in the hippocampus of the four groups of mice. **(I, J)** Representative images **(I)** and quantification **(J)** of Golgi staining in the hippocampus of the four groups of mice. I_(a)_ Scale bars = 200 μm. I_(b-e)_ Scale bars = 5 μm. DHT, dihydrotestosterone; Tfm, Testicular feminization mutation; PSD95, postsynaptic density protein 95; SYP, synaptophysin; ZIP9, Zrt-, Irt-like protein 9. (*P < 0.05, n = 6).

The IHC staining revealed significant inter group differences in the optical density of synaptic plasticity related proteins, such as PSD95 (F_(3,20)_ = 17.942, *P* < 0.05, η^2^ = 0.729), drebrin (F_(3,20)_ = 77.031, *P* < 0.05, η^2^ = 0.920), and SYP (F_(3,20)_ = 16.593, *P* < 0.05, η^2^ = 0.713). Compared to the WT+nc-RNAi group, the optical density of PSD95, drebrin, and SYP in the Tfm+nc-RNAi group decreased significantly. DHT supplementation increased the optical density of these hippocampal proteins in Tfm male mice, while the increase induced by DHT disappeared after hippocampal ZIP9 knockdown (**Figures 2A-D**). Western blot revealed non-significant differences in the expression of PSD95 (F_(3,20)_ = 5.833, *P* < 0.05, η^2^ = 0.467), drebrin (F_(3,20)_ = 7.278, *P* < 0.05, η^2^ = 0.552), and SYP (F_(3,20)_ = 12.693, *P* < 0.05, η^2^ = 0.656), as observed by IHC staining (**Figures 2E-H**).

Golgi staining results showed significant inter-group differences in the density of dendritic spines (F_(3,20)_ = 33.942, *P* < 0.05, η^2^ = 0.836). Compared to the WT+nc-RNAi group, the dendritic spine density in the hippocampi of the Tfm+nc-RNAi group decreased significantly. DHT supplementation increased the density of dendritic spines in Tfm male mice, but this increase was not observed after ZIP9 knockdown in the hippocampus (**Figures 2I, J** **)**.

### ZIP9 mediated the effects of DHT on the phosphorylation of ERK1/2 and eIF4E in Tfm male mice hippocampus and HT22 cells

**Figure 3:**
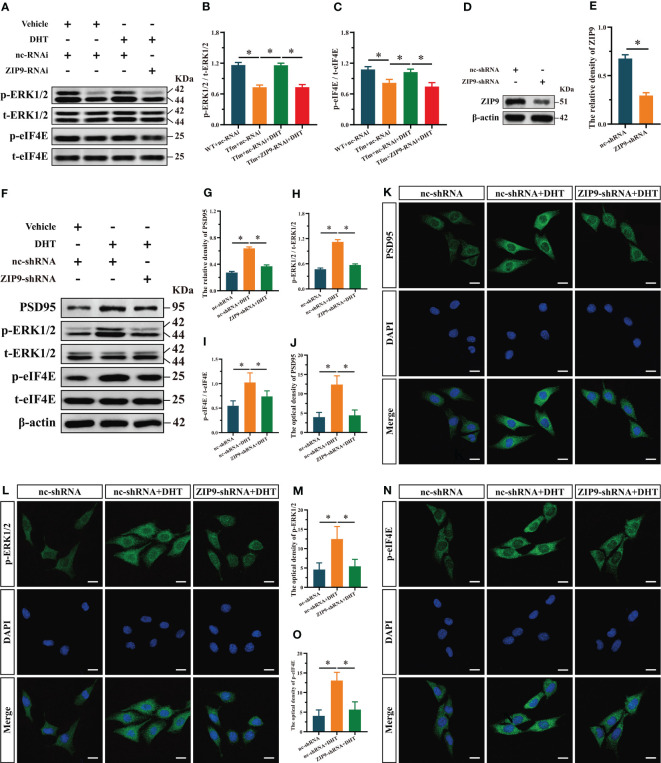
Effects of DHT on the phosphorylation of ERK1/2 and eIF4E in Tfm male mice and HT22 cells after ZIP9 knockdown. **(A–C)** Representative Western blot **(A)** and quantification **(B, C)** of the phosphorylation of ERK1/2 **(B)** and eIF4E **(C)** in the hippocampus of the four groups of mice. **(D, E)** The knockdown efficiency of ZIP9 protein in HT22 cells infected with ZIP9-shRNA or nc-RNA. **(F-I)** Representative Western blot **(F)** and quantification **(G-I)** of the expression of PSD95 **(G)**, and the phosphorylation of ERK1/2 **(H)**, eIF4E **(I)** in HT22 cells. **(J, K)** Representative IF staining images **(K)** and quantification **(J)** of PSD95 in HT22 cells. **(L, M)** Representative IF staining images **(L)** and quantification **(M)** of p-ERK1/2 in HT22 cells. **(N, O)** Representative IF staining images **(N)** and quantification **(O)** of p-eIF4E in HT22 cells after ZIP9 knockdown. Scale bars = 20 μm. IF, immunofluorescence; DHT, dihydrotestosterone; Tfm, Testicular feminization mutation; ERK1/2, Extracellular signal-regulated kinase ½; eIF4E, HT22, hippocampal neuron cells; ZIP9, Zrt-, Irt-like protein 9; PSD95, postsynaptic density protein 95. (*P < 0.05, n = 5).

To reveal the underlying mechanism, we studied the effect of DHT induced by ZIP9 on the phosphorylation of ERK1/2 and eIF4E in the hippocampi of Tfm male mice. Western blot revealed significant inter group differences in the phosphorylation of ERK1/2 (F_(3,16)_ = 143.584, *P* < 0.05, η^2^ = 0.964) and eIF4E (F_(3,16)_ = 31.446, *P* < 0.05, η^2^ = 0.855). Compared to the WT+nc-RNAi group, the phosphorylation of ERK1/2 and eIF4E in the Tfm+nc-RNAi group decreased significantly. DHT supplementation increased the phosphorylation of ERK1/2 and eIF4E in Tfm male mice, but this increase was not observed after ZIP9 knockdown in the hippocampus (**Figures 3A-C**).

To study the effects of DHT mediated by ZIP9 on the expression of PSD95 and the phosphorylation of ERK1/2 and eIF4E in HT22 cells, we constructed ZIP9 knockdown or overexpression HT22 stable cell lines by lentivirus infection. The expression of ZIP9 in the ZIP9-shRNA group was significantly lower than that in the nc-shRNA group (*t*_(8)_ = 17.814, *P* < 0.05, Cohen’s *d* = 25.193) (**Figures 3D, E** **)**; the expression of PSD95 (F_(2,12)_ = 550.836, *P* < 0.05, η^2^ = 0.989), phosphorylation of ERK1/2 (F_(2,12)_ = 402.259, *P* < 0.05, η^2^ = 0.985), and eIF4E (F_(2,12)_ = 14.350, *P* < 0.05, η^2^ = 0.705) in the nc-shRNA+DHT group were significantly higher than those in the nc-shRNA and ZIP9-shRNA+DHT groups (**Figures 3F-I**). IF staining showed that the optical density of PSD95 (F_(2,12)_ = 39.748, *P* < 0.05, η^2^ = 0.869), p-ERK1/2 (F_(2,12)_ = 17.002, *P* < 0.05, η^2^ = 0.739), and p-eIF4E (F_(2,12)_ = 33.090, *P* < 0.05, η^2^ = 0.847) in the nc-shRNA+DHT group were significantly higher than those in nc-shRNA and the ZIP9-shRNA+DHT groups (**Figures 3J-O**).

**Figure 4:**
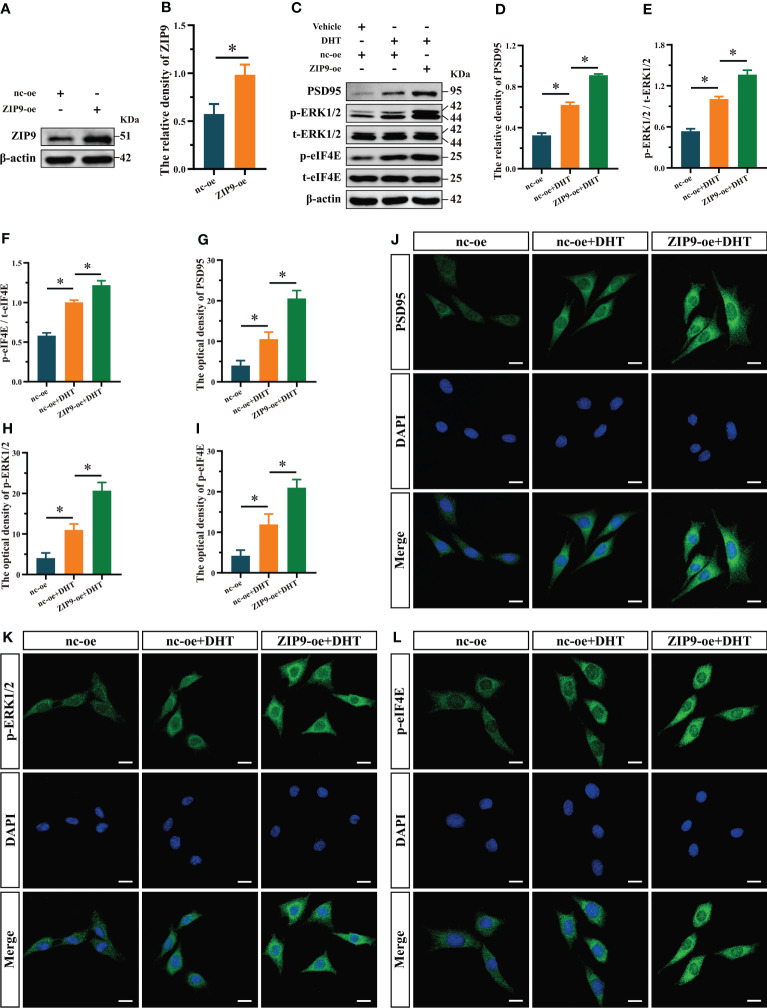
Effects of DHT on the expression of PSD95 and the phosphorylation of ERK1/2, eIF4E in HT22 cells after ZIP9 overexpression. **(A, B)** The overexpression efficiency of ZIP9 protein in HT22 cells infected with ZIP9-oe or nc-oe. **(C–F)** Representative Western blot **(C)** and quantification **(D–F)** of PSD95 **(D)**, and the phosphorylation of ERK1/2 **(E)** and eIF4E **(F)** in HT22 cells. **(G, J)** Representative IF staining images **(J)** and quantification **(G)** of PSD95 in HT22 cells. **(H, K)** Representative IF staining images **(K)** and quantification **(H)** of p-ERK1/2 in HT22 cells. **(I, L)** Representative IF staining images **(L)** and quantification **(I)** of p-eIF4E in HT22 cells. Scale bars = 20 μm. DHT, dihydrotestosterone; ERK1/2, Extracellular signal-regulated kinase ½; eIF4E, Eukaryotic translation initiation factor 4E; ZIP9, Zrt-, Irt-like protein 9; PSD95, postsynaptic density protein 95. (*P < 0.05, n = 5).

We observed the effects of DHT on the expression of PSD95 and phosphorylation of ERK1/2 and eIF4E in ZIP9-overexpression HT22 cells. The results of Western blot showed that the expression of ZIP9 in the ZIP9-oe group was significantly higher than that in the nc-oe group (*t*_(8)_ = -6.160, *P* < 0.05, Cohen’s *d* = -8.711, **Figures 4A, B** **)**; expression of PSD95 (F_(2,12)_ = 1003.038, *P* < 0.05, η^2^ = 0.994), phosphorylation of ERK1/2 (F_(2,12)_ = 385.322, *P* < 0.05, η^2^ = 0.985), and eIF4E (F_(2,12)_ = 291.648, *P* < 0.05, η^2^ = 0.980) in the nc-oe+DHT group were significantly higher than those in the nc-oe group, but lower than those in the ZIP9-oe+DHT group (**Figures 4C-F**). IF staining showed that the optical densities of PSD95 (F_(2,12)_ = 126.544, *P* < 0.05, η^2^ = 0.955), p-ERK1/2 (F_(2,12)_ = 132.102, *P* < 0.05, η^2^ = 0.957) and p-eIF4E (F_(2,12)_ = 86.879, *P* < 0.05, η^2^ = 0.935) in the nc-oe+DHT group were significantly higher than those in the nc-oe group, but lower than those in the ZIP9-oe+DHT group (**Figures 4G-L**).

### ZIP9 mediated the effects of DHT on the phosphorylation of ERK, eIF4E and expression of PSD95 in HT22 cells pretreated with SCH772984

**Figure 5:**
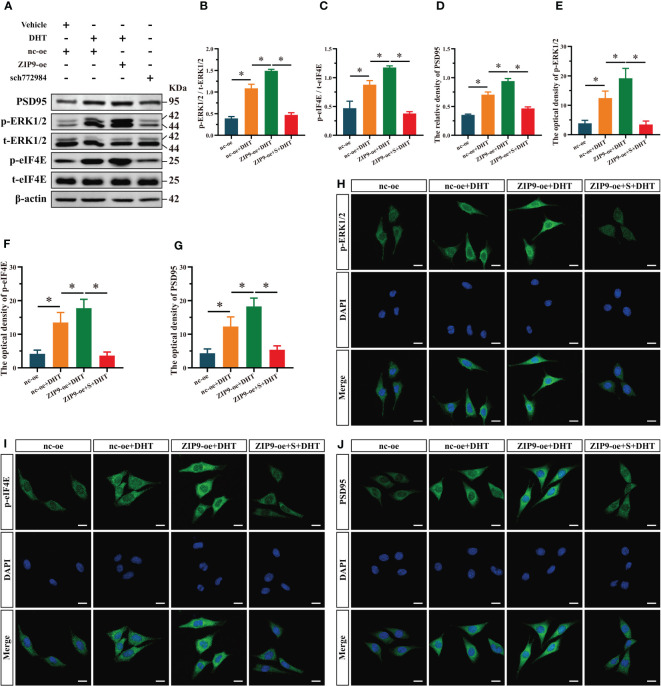
ZIP9 mediated the effects of DHT on the phosphorylation of ERK1/2, eIF4E and Expression of PSD95 after ZIP9 overexpression. **(A–D)** Representative Western blot **(A)** and quantification **(B–D)** of the phosphorylation of ERK1/2 **(B)**, eIF4E **(C)** and the expression of PSD95 **(D)** in HT22 cells pretreated with SCH772984. **(E, H)** Representative IF staining images **(H)** and quantification **(E)** of p-ERK1/2 in HT22 cells. **(F, I)** Representative IF staining images **(I)** and quantification **(F)** of p-eIF4E in HT22 cells. **(G, J)** Representative IF staining images **(J)** and quantification **(G)** of PSD95 in HT22 cells. Scale bars = 20 μm. DHT, dihydrotestosterone; ZIP9, Zrt-, Irt-like protein 9; PSD95, postsynaptic density protein 95, ERK1/2, Extracellular signal-regulated kinase ½; eIF4E, Eukaryotic translation initiation factor 4E. S, SCH772984. (*P < 0.05, n = 5).

We used SCH772984, a specific inhibitor of ERK1/2, to verify whether ERK1/2 is involved in the expression of PSD95 in HT22 cells induced by DHT through ZIP9. Western blot revealed significant inter group differences in the phosphorylation of ERK1/2 (F_(3,16)_ = 374.158, *P* < 0.05, η^2^ = 0.986), eIF4E (F_(3,16)_ = 124.188, *P* < 0.05, η^2^ = 0.959), and the expression of PSD95 (F_(3,16)_ = 274.867, *P* < 0.05, η^2^ = 0.981). The phosphorylation of ERK1/2 and eIF4E and the expression of PSD95 in the nc-oe+DHT group were higher than those in the nc-oe group and lower than those in the ZIP9-oe+DHT group, whereas these values for the ZIP9-oe+S+DHT group were lower than those for the ZIP9-oe+DHT group (**Figures 5A-D**). Consistent with the results of Western blot, IF staining demonstrated that the optical densities of p-ERK1/2 (F_(3,16)_ = 59.122, *P* < 0.05, η^2^ = 0.917), p-eIF4E (F_(3,16)_ = 55.533, *P* < 0.05, η^2^ = 0.912), and PSD95 (F_(3,16)_ = 48.888, *P* < 0.05, η^2^ = 0.902) were significantly different in all groups. The optical densities of p-ERK1/2, p-eIF4E, and PSD95 in the nc-oe+DHT group were higher than those in the nc-oe group and lower than those in the ZIP9-oe+DHT group, whereas those in the ZIP9-oe+S+DHT group were lower than those in the ZIP9-oe+DHT group (**Figures 5E-J**).

### ZIP9 mediated the effects of DHT on the phosphorylation of eIF4E and the Expression of PSD95 in HT22 cells pretreated with eFT508

**Figure 6:**
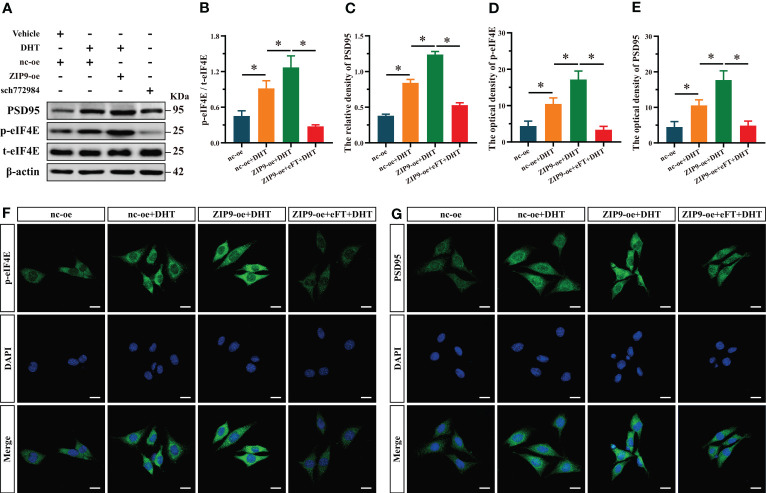
ZIP9 mediated the effects of DHT on the phosphorylation of eIF4E and the expression of PSD95 in HT22 cells pretreated with eFT508. **(A-C)** Representative Western blot **(A)** and quantification **(B, C)** of the Phosphorylation of eIF4E **(B)** and expression of PSD95 **(C)** in HT22 cells pretreated with eFT508. **(D, F)** Representative IF staining images **(F)** and quantification **(D)** of p-eIF4E in HT22 cells pretreated with eFT508. **(E, G)** Representative IF staining images **(G)** and quantification **(E)** of PSD95 in HT22 cells pretreated with eFT508. Scale bars = 20 μm. DHT, dihydrotestosterone; ZIP9, Zrt-, Irt-like protein 9; PSD95, postsynaptic density protein 95, ERK1/2, Extracellular signal-regulated kinase ½; eIF4E, Eukaryotic translation initiation factor 4E; eFT, eFT508. (*P < 0.05, n = 5).

Finally, we used eFT508, a specific inhibitor of eIF4E, to confirm whether eIF4E was involved in the expression of PSD95 in HT22 cells induced by DHT through ZIP9. Western blot revealed significant inter group differences in the phosphorylation of eIF4E (F_(3,16)_ = 320.397, *P* < 0.05, η^2^ = 0.984) and the expression of PSD95 (F_(3,16)_ = 497.501, *P* < 0.05, η^2^ = 0.989). The phosphorylation of eIF4E and the expression of PSD95 in the nc-oe+DHT group were higher than those in the nc-oe group and lower than those in the ZIP9-oe+DHT group, whereas those in the ZIP9-oe+eFT+DHT group were lower than those in the ZIP9-oe+DHT group (**Figures 6A-C**). The IF staining demonstrated significant inter group differences in the optical densities of p-eIF4E (F_(3,16)_ = 74.668, *P* < 0.05, η^2^ = 0.933) and PSD95 (F_(3,16)_ = 59.422, *P* < 0.05, η^2^ = 0.918. The optical densities of p-eIF4E and PSD95 in the nc-oe+DHT group were higher than those in the nc-oe group and lower than those in the ZIP9-oe+DHT group, whereas these values for the ZIP9-oe+eFT+DHT group were lower than those for the ZIP9-oe+DHT group (**Figures 6D-G**).

### ZIP9 mediated the effects of DHT on learning and memory of APP/PS1 male mice

**Figure 7:**
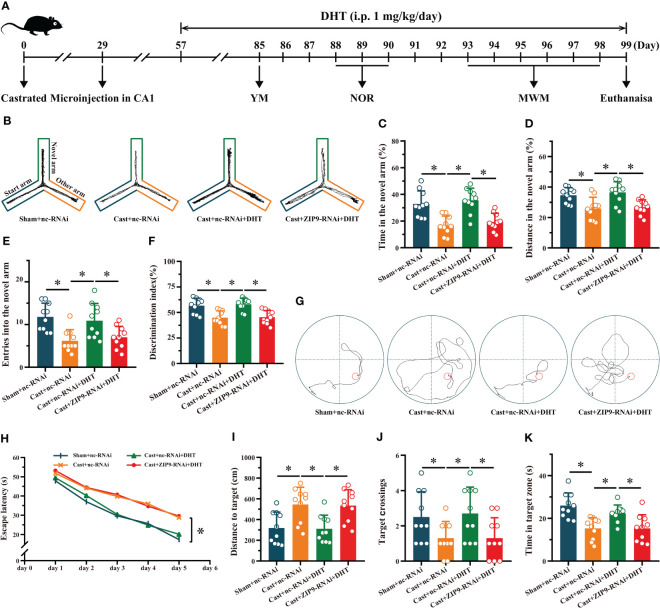
ZIP9 mediated the effects of DHT on learning and memory of APP/PS1 male mice. **(A)** Experimental procedure. Mice were treated with castration, microinjections in CA1, DHT (i.p. 1 mg/kg/day), and behavioral tests. **(B)** Representative trajectories of the YM (test). **(C–E)** YM was performed to test for spatial reference memory. **(F)** NOR was performed to assess memory retention. **(G)** Representative trajectories of the MWM (the 5th day). **(H-K)** MWM was used to test spatial memory and long-term memory. DHT, dihydrotestosterone; YM, Y-maze test; NOR, novel object recognition test; MWM, Morris water maze. (*P < 0.05, n = 10).

We verified the effect of DHT induced by ZIP9 on the behavior of APP/PS1 male mice (**Figure 7A**). The YM test showed significant inter group differences in the percentage of time spent (F_(3,36)_ = 13.241, *P* < 0.05, η^2^ = 0.525), the percentage of distance travelled (F_(3,36)_ = 7.551, *P* < 0.05, η^2^ = 0.386), and the number of entries (F_(3,36)_ = 7.675, *P* < 0.05, η^2^ = 0.390) in the ovel arm. The values of above parameters for Cast+nc-RNAi group were significantly lower than those for the sham+nc-RNAi and Cast+nc-RNAi+DHT groups, whereas the values for Cast+nc-RNAi+DHT group were higher than those for the Cast+ZIP9-RNAi+DHT group (**Figures 7B-E**).

The NOR test showed a significant inter group differences in DI (F_(3,36)_ = 11.338, *P* < 0.05, η^2^ = 0.486). The DI of the Cast+nc-RNAi group was significantly lower than that of the sham+nc-RNAi group. DHT supplementation increased the DI of castrated APP/PS1 mice, while this increase disappeared after ZIP9 knockdown in the hippocampus (**Figure 7F**).

In the Morris water navigation task, there were significant inter group differences in the escape latency at 1-5 days (F_(3,36)_ = 4.307, *P* < 0.05, η^2^ = 0.264) and the distance to the target on the 5th day (F_(3,36)_ = 7.271, *P* < 0.05, η^2^ = 0.377). The values of above parameters for the Cast+nc-RNAi group were significantly higher than those of the Sham+nc-RNAi and Cast+nc-RNAi+DHT groups, whereas the values for the Cast+nc-RNAi+DHT group were lower than those for the Cast+ZIP9-RNAi+DHT group. The subsequent spatial probe trial demonstrated significant differences in the number of target crossings (F_(3,36)_ = 3.803, *P* < 0.05, η^2^ = 0.241) and time in the target zone (F_(3,36)_ = 9.821, *P* < 0.05, η^2^ = 0.450). The number of target crossings and time in the target zone of the Cast+nc-RNAi group were lower than those of the sham+nc-RNAi and Cast+nc-RNAi+DHT groups; however, those of the Cast+nc-RNAi+DHT group were lower than those of the Cast+ZIP9-RNAi+DHT group (**Figures 7G-K**).

### ZIP9 mediated the effects of DHT on hippocampal PSD95, Drebrin, SYP protein and dendritic spine density of APP/PS1 male mice

**Figure 8:**
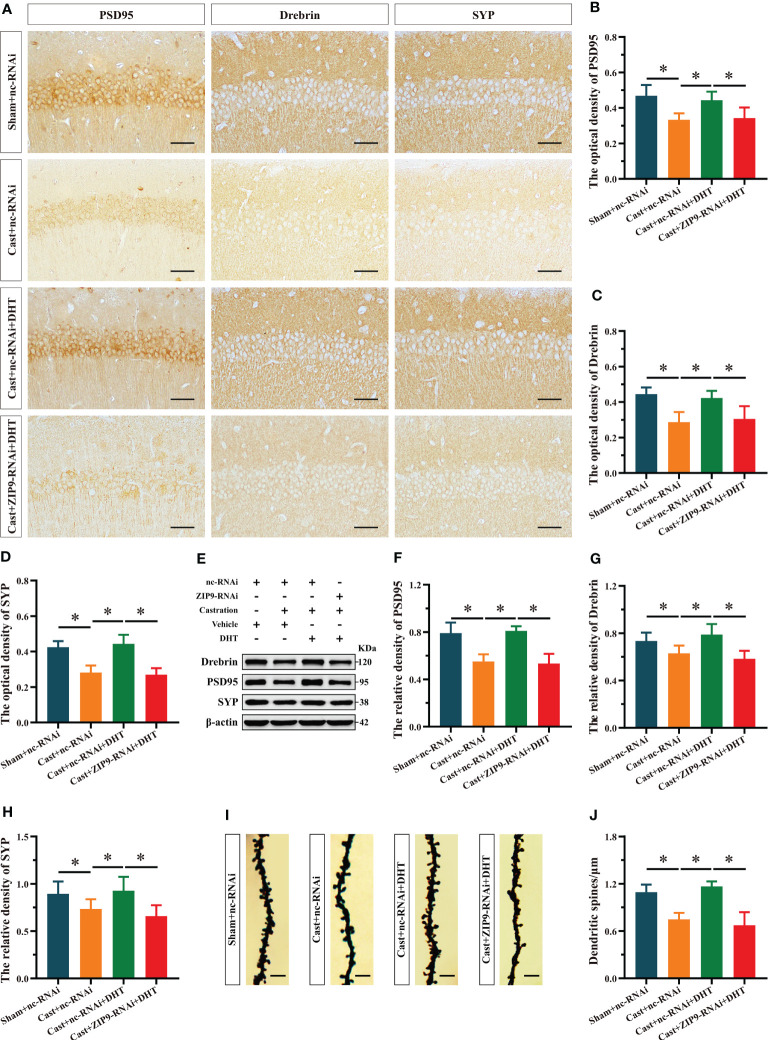
ZIP9 mediated the effects of DHT on hippocampal PSD95, drebrin, SYP protein and dendritic spine density in APP/PS1 male mice. **(A-D)** Representative IHC images **(A)** and quantification **(B–D)** of PSD95 **(B)**, drebrin **(C)**, and SYP **(D)** in the hippocampal CA1 region of the four groups of mice. Scale bars = 50 μm. **(E–H)** Representative Western blot **(E)** and quantification **(F–H)** of PSD95 **(F)**, drebrin **(G)**, and SYP **(H)** in the hippocampus of the four groups of mice. **(I, J)** Representative images **(I)** and quantification **(J)** of Golgi staining in the hippocampus of the four groups of mice; Scale bars = 5 μm. DHT, dihydrotestosterone; ZIP9, Zrt-, Irt-like protein 9; PSD95, postsynaptic density protein 95; SYP, synaptophysin. (*P < 0.05, n = 6).

After the behavioral experiments, we studied the effects of DHT mediated by ZIP9 on the expression of PSD95, drebrin, SYP, and the density of dendritic spines in the hippocampi of APP/PS1 male mice. The IHC staining revealed significant inter group differences in the optical density of PSD95 (F_(3,20)_ = 10.479, *P* < 0.05, η^2^ = 0.611), drebrin (F_(3,20)_ = 12.782, *P* < 0.05, η^2^ = 0.657) and SYP (F_(3,20)_ = 30.519, *P* < 0.05, η^2^ = 0.821). Compared with the Sham+nc-RNAi group, the optical density of PSD95, drebrin, and SYP in the Cast+nc-RNAi group decreased significantly. The DHT supplementation increased the optical density of these proteins in castrated APP/PS1 mice, while the increase vanished after ZIP9 knockdown in the hippocampi (**Figures 8A-D**). Western blot revealed similar results for the expression of PSD95 (F_(3,20)_ = 27.055, *P* < 0.05, η^2^ = 0.802) and drebrin (F_(3,20)_ = 9.642, *P* < 0.05, η^2^ = 0.591) and SYP (F_(3,20)_ = 6.262, *P* < 0.05, η^2^ = 0. 484) in the hippocampi as observed by IHC staining (**Figures 8E-H**).

Golgi staining results showed a significant inter group differences in the density of dendritic spines (F_(3,20)_ = 30.471, *P* < 0.05, η^2^ = 0.820). Compared to the Sham+nc-RNAi group, the dendritic spine density in the hippocampi of the Cast+nc-RNAi group decreased significantly. DHT supplementation increased dendritic spine density in castrated APP/PS1 mice, but this increase was not observed after ZIP9 knockdown in the hippocampus (**Figures 8I, J** **)**.

### ZIP9 mediated the effects of DHT on the phosphorylation of ERK1/2 and eIF4E in APP/PS1 mice hippocampus

**Figure 9:**
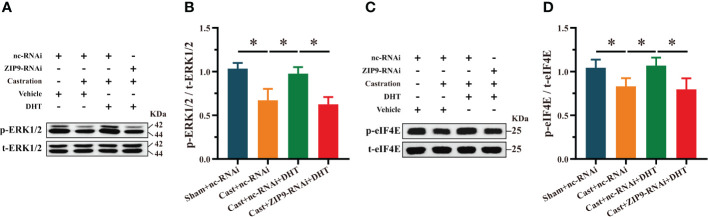
ZIP9 mediated the effects of DHT on the phosphorylation of ERK1/2 and eIF4E in APP/PS1 mice hippocampus. **(A, B)** Representative Western blot **(A)** and quantification **(B)** of the phosphorylation of ERK1/2 in the hippocampus of the four groups of mice. **(C, D)** Representative Western blot **(C)** and quantification **(D)** of the phosphorylation of eIF4E in the hippocampus of the four groups of mice. DHT, dihydrotestosterone; ZIP9, Zrt-, Irt-like protein 9; ERK1/2, Extracellular signal-regulated kinase ½; eIF4E, Eukaryotic translation initiation factor 4E. (*P < 0.05, n = 6).

We studied the effect of DHT mediated by ZIP9 on the phosphorylation of ERK1/2 and eIF4E in the hippocampi of APP/PS1 mice. Western blot revealed significant inter group differences in the phosphorylation levels of ERK1/2 (F_(3,20)_ = 31.214, *P* < 0.05, η^2^ = 824) and eIF4E (F_(3,20)_ = 13.242, *P* < 0.05, η^2^ = 0. 665). Compared to the Sham+nc-RNAi group, the phosphorylation of ERK1/2 and eIF4E in the Cast+nc-RNAi group decreased significantly. DHT supplementation improved the phosphorylation of ERK1/2 and eIF4E in castrated APP/PS1 mice, while improvement was not observed after ZIP9 knockdown in the hippocampus (**Figures 9A-D**).

## Discussion

The effect of androgens was previously thought to be mediated by AR. Androgens bind to intracellular AR and form a receptor-ligand complex, which is transferred into the nucleus to regulate target genes and exert biological effects. In addition to the classic AR, recent studies have found that androgen can also exert effects through other binding sites, such as GPRC6A (30, 31), TRPM8 (32), OXER1 (33) and ZIP9 (28). As a member of the ZIP family, ZIP9 participates in the transport of Zn**^2+^** from extracellular to intracellular matrix (19). It can also bind to androgens and further couple with the G protein to exert biological effects. In cancer studies, androgens have been found to bind to ZIP9 and exert biological effects. Bulldan et al. (22) found that ZIP9 mediates testosterone-induced migratory activity of metastatic prostate cancer cells. Chen et al. (23) suggested that DHT may increase the migration and invasion of AR-negative bladder cancer cells *via* ZIP9, thus promoting the progression of muscle-invasive bladder cancer. Thomas et al. (24) reported that the expression of ZIP9 was upregulated in breast and prostate cancer tissues, and androgen promoted the apoptosis of breast cancer MDAMB-468 cells and prostate cancer PC-3 cells through ZIP9. The authors also noted that ZIP9 was a potential therapeutic target in breast and prostate cancer. ZIP9 is also widely expressed in normal tissues and cells, such as the testis, pancreas, heart, prostate and brain (24). In a study of Sertoli cells, Bulldan et al. (34) found that testosterone can promote the expression of the tight junction protein claudin and the formation of tight junctions through ZIP9, highlighting the importance of this mechanism in male reproductive function. Converse et al. (35) found that androgen regulated stage-dependent pro- and anti-apoptosis in teleost ovaries through ZIP9 by activating different G proteins. They also demonstrated *via* another study (36) that ZIP9 mediated androgen effect in promoting the proliferation of vascular endothelial cells. Malviya et al. (37) suggested that testosterone promotes mineralization in human osteoblastic SAOS-2 cells and myogenesis in mouse myogenic L6 cells through ZIP9. Although our previous studies have shown that T-BSA rapidly increases the expression of PSD95 protein in HT22 cells through ZIP9, it is not yet known whether ZIP9 is involved in learning and memory as an androgen binding site in the hippocampus. The present study used AR-deficient Tfm male mice with learning and memory impairments to study the effects of androgens mediated by ZIP9 on hippocampal learning and memory. The results showed that ZIP9 was expressed in the hippocampus of WT and Tfm male mice with no significant differences between the groups, suggesting that ZIP9 did not directly affect the learning and memory of Tfm mice. However, this did not exclude the possibility that ZIP9, as an androgen-binding site, affects learning and memory in mice.

We used androgen supplementation and hippocampal ZIP9 knockdown models to determine whether androgen affects learning and memory of Tfm male mice through ZIP9. Testosterone can be partially converted by aromatase into estrogen (38, 39), which can affect synaptic plasticity in the hippocampus of mice (40, 41). To avoid this, we used non-aromatized DHT. Behavioral experiments showed that DHT supplementation significantly improved learning and memory in Tfm male mice, but this improvement was inhibited after hippocampal ZIP9 knockdown. This suggests that DHT improved the learning and memory of Tfm male mice through ZIP9. After behavioral experiments, we evaluated hippocampal synaptic plasticity in Tfm male mice since it plays an important role in learning and memory. PSD95 is essential for synaptic plasticity of the nervous system as a scaffold protein in the postsynaptic structure, PSD95-knockout mice showed long-term potentiation, long-term depression impairment, and significant spatial learning and memory impairment in behavioral tests (42). As an important actin-binding protein widely distributed in dendritic spines, drebrin can regulate synaptic plasticity and affect cognitive function by combining with F-actin to promote dendritic spine maturation (36). SYP is important for synaptic plasticity and cognitive function and its content can be used as an index to evaluate the number, density, and transmission efficiency of synapses (43). Dendritic spines are the main sites of synapse formation. Loss of dendritic spines is closely related to a decline in cognitive ability (44, 45). Therefore, we investigated the expression level of synaptic proteins PSD95, drebrin, SYP and the density of dendritic spines to observe the effect of DHT supplementation on synaptic plasticity in Tfm male mice. The results showed that the expression of these components, as well as density of dendritic spines were significantly lower than in WT male mice, with a significant increase after DHT supplementation. To explore how DHT works, we knocked down hippocampal ZIP9 in Tfm male mice and found that the increase in the expression of PSD95, drebrin, and SYP, and the density of dendritic spines induced by DHT disappeared. Combined with the results of the behavioral experiments, we postulate that DHT improves hippocampal synaptic plasticity in Tfm male mice through ZIP9, thereby improving their learning and memory.

The extracellular-signal-regulated kinase (ERK) may be a key downstream signal molecule of ZIP9-mediated androgen effect. Profaska-Szymik et al. (46) found that senescence driven by androgens *via* ZIP9 in regressed vole testes has a functional link with ERK. Other studies (20, 27, 47–51) have also found ZIP9 mediated androgen biological effects through ERK pathway. In the nervous system, ERK plays a vital role in synaptic plasticity, learning, and memory (52–55). The phosphorylation of ERK1/2 can enhance the uncoupling of nNOS-PSD95 in the mouse hippocampus, increase the expression of PSD95 protein, promoting memory retrieval et al. (56). The cap-binding translation initiation factor eIF4E, cooperating with proteins such as helicase eIF4A and scaffolding protein eIF4G binds to mRNA, allowing the recruitment of ribosomes and translation initiation (57, 58). Gindina et al. (59) found upregulation of eIF4E in dendritic spines during memory formation in adult male Sprague-Dawley rats. These results are consistent with those of our study. The phosphorylation of ERK1/2 and eIF4E in the hippocampus of Tfm male mice was significantly lower than that in WT male mice; DHT supplementation significantly increased these levels, suggesting that there was abnormal phosphorylation of ERK1/2 and eIF4E in Tfm male mice, and that DHT could improve this abnormality. To find out whether ZIP9 mediated this effect of DHT, we knocked down hippocampal ZIP9 in Tfm male mice and found that the beneficial effect of DHT were inhibited. These results were also confirmed at a cellular level. This confirmed that increase in the expression of PSD95 and phosphorylation of ERK1/2 and eIF4E in HT22 cells induced by DHT were inhibited after ZIP9 knockdown and enhanced after ZIP9 overexpression.

Although we found that DHT promoted the phosphorylation of ERK1/2, eIF4E and the expression of PSD95 through ZIP9, the mechanism still required experimental verification. We found that pretreatment with SCH772984, a specific inhibitor of ERK1/2, significantly inhibited the phosphorylation of ERK1/2, eIF4E and expression of PSD95. Pretreatment with eFT508, a specific inhibitor of eIF4E, significantly inhibited the phosphorylation of eIF4E and expression of PSD95 in ZIP9-overexpression HT22 cells. These results are consistent with those of other studies showing that ERK1/2 activation can regulate synaptic protein synthesis by phosphorylating eIF4E, which subsequently affects synaptic plasticity (60, 61). Combined with animal and cell experiments, it was concluded that ZIP9 mediated the effects of DHT on improving the expression of synaptic plasticity-related proteins and dendritic spine density in the hippocampus of Tfm male mice through the ERK1/2-eIF4E pathway, thus improving learning and memory.

The serum level of total or free testosterone in AD patients is significantly lower than that in normal elderly men (62), and the decreased level of testosterone *in vivo* leads to cognitive decline, which was measured by learning and memory (63). Studies have reported that cognitive function of AD patients improved to varying degrees after testosterone replacement therapy (64). This suggests that androgen decline may be a risk factor for AD in older men. Therefore, castration surgery or androgen supplement therapy are often used to intervene in AD animal models to study the pathological mechanism of androgen regulation of AD cognitive impairment (65–68). APP/PS1 is a double-transgenic mouse model that overproduces Aβ and is often used to study the mechanisms of AD neuropathology. Aβ plaques appear in the cerebral cortex at approximately 4 months of age and in the hippocampus at 6 months of age, and increase in size and number with age (69, 70). Obvious learning and memory deficits emerge at 6-10 months old (71, 72). This study found that castration significantly impaired the learning and memory of APP/PS1 mice and decreased the expression of PSD95, drebrin, SYP, and dendritic spine density. Supplementation with DHT ameliorated the adverse effects of castration. To verify whether the improvement induced by DHT in castrated mice depends on ZIP9, we knocked down hippocampal ZIP9 in castrated APP/PS1 mice and found that the improvement of learning and memory, the expression of PSD95, drebrin, SYP, and dendritic spine density induced by DHT were significantly suppressed. Castration significantly decreased the phosphorylation of ERK1/2 and eIF4E in the hippocampus of APP/PS1 mice, and DHT supplementation could improve this deficiency state. To determine the effect of DHT on the phosphorylation of ERK1/2 and eIF4E through ZIP9, we knocked down hippocampal ZIP9 in castrated APP/PS1 mice and found that the phosphorylation of ERK1/2 and eIF4E was significantly downregulated. This suggests that ZIP9 mediates the effects of DHT on the expression of synaptic plasticity-related proteins and dendritic spine density in the hippocampi of APP/PS1 mice through the ERK1/2-eIF4E pathway and affects their learning and memory.

The ZIP9 has been described as a membrane androgen receptor (19, 20, 26, 28, 46, 48, 73–75). Thomas et al. (24) found ZIP9 in the perinuclear and plasma membrane of MDA-MB-468 cells, while some studies also detected it in the reverse Golgi network (76). In addition, since membrane permeable DHT was used in this study, the current data do not support the conclusion that DHT played a role only through ZIP9 located in the cell membrane. However, our experimental results confirmed that DHT could regulate hippocampal synaptic plasticity through ZIP9, thus affecting learning and memory, which was consistent with our research theme.

However the findings of this study have to be seen in light of some limitations. Firstly, considering that the topic of this study is the effects of DHT mediated by ZIP9 on hippocampal synaptic plasticity and learning and memory, we did not detect hippocampal Aβ in APP/PS1 mice, although senile plaque formed by excessive deposition of Aβ is one of the pathological features of AD. Therefore, we are unable to know the effects of DHT mediated by ZIP9 on hippocampal pathology in APP/PS1 mice. Secondly, ZIP9 has both androgen signaling and zinc transport functions, and the mechanism of zinc ion on learning and memory is still unclear. Since this study focused on ZIP9-mediated androgen influence on synaptic plasticity and learning memory in mice, its zinc transport function was not investigated. Finally, only male mice were used in this study, and the results of this study may not be applicable to female animals because of gender differences.

Based on the above experiments, we concluded that ZIP9 mediated the effects of DHT on improving hippocampal synaptic plasticity-related proteins and dendritic spine density in Tfm male mice through the ERK1/2-eIF4E pathway, improving learning and memory. DHT can also affect learning and memory in castrated APP/PS1 mice through this mechanism. This study provides important experimental data for further research into the use of androgen supplementation in Alzheimer’s Disease to improve learning and memory.

## Data availability statement

The original contributions presented in the study are included in the article/**Supplementary Material**. Further inquiries can be directed to the corresponding author/s.

## Ethics statement

The animal study was reviewed and approved by Hebei Medical University Laboratory Animal Welfare and Ethics Committee.

## Author contributions

HXC and SL designed the research, writing-review, editing, project administration. LS and HC performed the experimental phase, methodology, data curation, writing-original draft. DQ, BZ, FG, YZ and CW contributed toward investigation, and visualization. All authors contributed to the article and approved the submitted version.
