# Supplementary material for: ZIP9 mediates the effects of DHT on learning, memory and hippocampal synaptic plasticity of male Tfm and APP/PS1 mice
Source: Front Endocrinol (Lausanne). 2023 May 25;14:1139874. doi: 10.3389/fendo.2023.1139874 (PMC10248430; doi:10.3389/fendo.2023.1139874)
Supplement: Supplementary file 2 [file Table_1.docx]

**KEY RESOURCES TABLE**

| Reagent or resource | Source | Identifier |
| --- | --- | --- |
| Antibodies | | |
| rabbit rabbit anti-ZIP9 antibody | GeneTex | cat#: GTX31817 |
| anti-phospho-ERK1/2 | cell signaling | cat#: 9101 |
| mouse anti-ERK1/2 | cell signaling | cat#: 9107 |
| rabbit anti-phospho-eIF4E | Abcam | cat#: ab76256 |
| mouse anti-eIF4E (total) | Abcam | cat#: ab171091 |
| rabbit anti-PSD95 | Abcam | cat#: ab18258 |
| rabbit anti-synaptophysin | Abways | cat#: CY5273 |
| rabbit anti-drebrin | Proteintech | cat#: 10260-1-AP |
| mouse anti-β-actin | Proteintech | cat#: CL594-60008PH |
| goat anti-rabbit fluorescent secondary antibody | Rockland | cat#: 611145002 |
| goat anti-mouse secondary antibody | Rockland | cat#: 610144002 |
| donkey anti-rabbit fluorescent secondary antibody | Invitrogen | cat#: A21207 |
| Bacterial and Virus Strains | | |
| ZIP9-shRNA(GV248) | Genechem | GIEL0106631 |
| ZIP9-overexpression(GV722) | Genechem | GOSL0301779 |
| AAV9-ZIP9-RNAi(GV478) | Genechem | GIDV0190942 |
| Chemicals, Peptides, and Recombinant Proteins | | |
| DHT | Tokyo Chemical Industry | cat#: A0462 |
| SCH772984 | Selleck | cat#: S7101 |
| Tomivosertib (eFT508) | MCE | cat#: HY-100022 |
| DMSO | Tokyo Chemical Industry | cat#: D0798 |
| DAPI | Solarbio | cat#: C0065 |
| DMEM/F12 | Procell | cat#: PM150316 |
| isoflurane | RWD | cat#: R510-22-10, |
| Experimental Models: Cell Lines/Mice | | |
| HT22 cells | Procell | cat#: PM150316 |
| Tfm mice | the Jackson Laboratory | stock#: 000569 |
| C57BL/6J mice | Vital River | N/A |
| APP/PS1 mice | Vital River | N/A |
| Critical Commercial Assays | | |
| Golgi staining kit | GENMED | cat#: GMS80020.1 |
| rabbit SP detection kit | ZSGB-BIO | cat#: SP-9001 |
| Software and Algorithms | | |
| ImageJ (FIJI) | NIH | N/A |
| SPSS 26.0 | IBM | N/A |
| G*Power | www.gpower.hhu.de | N/A |
